# Involving Carers in Therapy for Adults With Intellectual Disabilities: A Systematic Review of Client, Carer and Therapist Perspectives

**DOI:** 10.1111/jar.70152

**Published:** 2025-11-19

**Authors:** Charlotte Farnsworth, Thomas Schröder

**Affiliations:** ^1^ Trent Doctorate in Clinical Psychology, Mental Health and Clinical Neurosciences, School of Medicine University of Nottingham Nottingham UK; ^2^ Nottinghamshire Healthcare NHS Foundation Trust Nottingham UK

**Keywords:** carer involvement, intellectual disability, meta‐ethnography, psychological therapy

## Abstract

**Background:**

Adaptations are frequently reported when delivering therapy to people with an intellectual disability, including the involvement of carers. This review aimed to understand the experience of carer involvement from the perspective of the person, therapist and caregiver.

**Methods:**

A systematic search of six databases and of reference lists was conducted. The quality of the included studies was assessed using an adapted version of the Critical Appraisal Skills Programme qualitative checklist. Data was synthesised using meta‐ethnography.

**Results:**

Sixteen studies were included but their quality varied. Five third‐order constructs were identified: safety, therapeutic process, relationships, personal impact on carer and improving the experience.

**Conclusion:**

Findings suggest that carer involvement can be a positive experience and aid the therapy process. However, this depended on several factors including carer motivation and consistency. Confidentiality issues were highlighted. Limitations of studies included poor reporting on participant demographics and small samples. Future research and clinical implications are suggested.

**Summary**

- Involving carers in therapy for someone with an intellectual disability is a mixed experience.
- It can be better when it is always the same carer and when the carer is motivated. People with an intellectual disability also sometimes want time alone with their therapist.
- The results are important as they can tell us the best way of involving carers to make therapy better for people with an intellectual disability.
- However more research is still needed to see when and how involving carers in therapy is a good experience.

## 1 Introduction

People with intellectual disabilities are more likely to experience poor mental health than those without (Hughes‐McCormack et al. 2017). Cognitive deficits seen in those with an intellectual disability may make engaging in psychological therapy difficult. Despite this, research shows that this population is able, and has the skills, to engage in therapy (Taylor et al. 2008). However, adaptations may be required to address the cognitive deficits (Lindsay et al. 2013).

The National Institute for Health and Clinical Excellence (NICE) highlights the importance of tailoring an intervention to the person's level of understanding and strengths whilst considering their impairments or communication needs (NICE 2016). There are a number of ways in which therapy can be adapted to suit the needs of people with an intellectual disability depending on the therapeutic model (Beail 2017). This includes the therapist working within the client's communication abilities, using flip charts or visual aids, having more but shorter sessions, repeatedly checking for understanding and involving carers or family members.

Past literature reviews have reported on the frequency and nature of therapy adaptations, showing that carer involvement is frequently reported (Surley and Dagnan 2019; Tapp et al. 2023). Carer involvement has been suggested for a range of adapted interventions including eye‐movement desensitisation and reprocessing therapy (EMDR; Schipper‐Eindhoven et al. 2024) and behavioural activation (Jahoda et al. 2017). Ultimately, NICE guidelines recommend collaborating with the individual and their caregivers, who may be family, friends or paid support staff, to practice skills between sessions (NICE 2016). Carers may be informal (typically the person's family or friends), or formal (paid support staff). In the literature, carers have been termed ‘supporters’, ‘informants’ or ‘support staff’. For consistency, the terms ‘carer’ or ‘caregiver’ will be used in this review.

Carer involvement has been considered an important adaptation in care for people with an intellectual disability. Within physical healthcare, previous research has suggested that where carers feel understood and involvement has been positive, client care and outcomes are improved whereas patient care is compromised due to inadequate carer involvement (Tuffrey‐Wijne et al. 2013). Similar reports have been subsequently reported highlighting that when carers are effectively involved in care for people with an intellectual disability, the quality of the person's care is improved (Almas et al. 2025). This highlights the importance of exploring ways of optimising carer involvement.

It has been found that adults with an intellectual disability who attended a cognitive‐behaviour group intervention with their carer were more likely to benefit from the group than those without (Rose et al. 2005). This reinforces that as well as in physical healthcare, there may be improved outcomes through carer involvement in mental health interventions. Benefits of involving a carer have been identified and include supporting with homework, enabling communication between client and therapist, supporting engagement and maintaining changes post‐therapy (Dagnan et al. 2023; Willner 2006). Furthermore, a previous review found that carer‐led health interventions improved mental health outcomes (Hithersay et al. 2014).

Despite this, benefits may also be limited based on carer job satisfaction, and the willingness and knowledge of the carer themselves (Willner 2006). These barriers may need to be addressed before benefits may come from carer involvement. Furthermore, the evidence suggests that carer involvement is beneficial to patient outcomes, leading to the recommended guidance. Nevertheless, there is little insight into how this is perceived by those involved in the therapy. When exploring other populations, research has found that involving parents as co‐therapists may lead to frustration and communication difficulties (Lundkvist‐Houndoumadi et al. 2015) suggesting that involvement, whilst recommended, may also be detrimental to relationships. However, this study was looking into a child population and actually little is known about how carer involvement is perceived within an intellectual disability population.

People with an intellectual disability have previously been excluded from research but recently there has been an increase in promoting their participation (Beighton et al. 2019). Studies have suggested adaptations that can aid their involvement, such as using visual aids during interviews, and offering the person choice in date, time and location (Drozd et al. 2021). To give a voice to all parties involved in the therapy room, the current review considers the perspectives of the person, carer and therapist to gain a holistic understanding of the experience of carer involvement.

Past reviews have shown that carer involvement is frequently reported as an adaptation in therapy for adults with an intellectual disability and has the potential to contribute to improved therapeutic outcomes. It is therefore recommended by national guidance. However, to our knowledge, no previous review has explored the evidence base relating to the actual experience of all concerned. Consequently, this systematic review addressed the questions of how carer involvement in therapy is received, and when it may be most useful. The results may be useful in informing future policy or guidance for clinicians to maximise potential benefits from carer involvement.

## 2 Method

### 2.1 Meta‐Ethnography

Meta‐synthesis attempts to combine results from related qualitative studies (Walsh and Downe 2005) and can make previously overlooked qualitative data visible (Horton 2020). Within healthcare, it aims to improve patient care by increasing awareness and knowledge of patient experience (Toye et al. 2014).

A particular approach to synthesising qualitative research is meta‐ethnography (Noblit and Hare 1988), which seeks to establish where findings agree and disagree by ‘translating’ concepts and themes across different studies into one another to arrive at more comprehensive interpretations. Meta‐ethnography is useful for researchers and policy makers in making sense of complex phenomena and identifying patterns and variations within findings. If done well it may contribute to novel understandings of complex issues (Luong et al. 2023). As the current review aimed to explore the complex pattern of the experiences of different groups to contribute to new understandings, this synthesis method was deemed most appropriate.

This review followed the seven phases for meta‐ethnography (Noblit and Hare 1988) and followed the eMERGE guidance for meta‐ethnography reporting (France et al. 2019). PRISMA guidance (Page et al. 2021) for conducting systematic reviews was also followed to ensure the quality of the current review.

When conducting meta‐ethnographies, participant quotes are considered first‐order constructs whilst the author interpretations or themes are considered second‐order constructs. Third‐order constructs are therefore the themes identified through synthesis (Britten et al. 2002). Within meta‐ethnography, synthesis can occur in three ways (Sattar et al. 2021):

– Reciprocal translation (how studies are similar).
– Refutational translation (how studies are different).
– Line of argument synthesis (bringing it all together).

### 2.2 Search Strategy

An initial scoping review was carried out to ascertain the state of the existing literature. This showed a lack of research specifically aimed at gathering data on the experience of carer involvement in therapy; instead, any references to carer involvement were often brief comments within larger discussions on the acceptability or feasibility of an intervention. Therefore, the search strategy was designed to be open to capture any relevant data.

A systematic literature search of five major databases (PsychINFO, Scopus, Web of Science, CINAHL and Medline) was completed on 21 June 2024. In addition, the Open Dissertation database was searched for unpublished theses. All database searching and screening was carried out by the first author.

Search terms were developed in collaboration with a librarian specialising in Psychology. Search terms were: (“intellectual dis*” OR idd OR “intellectual developmental dis*” OR “learning dis*”) AND (carer* OR caregiver OR “care‐giver” OR “care giver” OR “family involvement” OR “family assisted” OR “family led” OR “parent assisted” OR “parent led” OR supporter* OR “support worker*” OR friend OR “supportive other*”) AND (“mental health” OR psycholog* OR therap* OR psychotherap*) AND (view* OR opinion* OR perception* OR attitude* OR experience* OR qualitative OR “mixed‐method*” OR “mixed method*”). Relevant keywords and medical subject heading (MESH) terms were selected for the appropriate databases.

Results from the searches were imported to the online systematic review management software, Covidence, which was used as a tool to manage search results. An artificial intelligence (AI) tool within Covidence was used to supplement the first phase of removing duplicates. This was checked by the first author and further copies were removed. After the removal of duplicates, titles and abstracts were screened for inclusion and exclusion criteria (see Table 1). Full texts were then read to determine eligibility. AI was not used to assist in determining eligibility. There were no thresholds set in terms of how old the research was, nor the language of the paper. From the papers that were identified as relevant, reference lists were reviewed to further broaden the scope of the search.

**TABLE 1:** Inclusion and exclusion criteria.

	Inclusion	Exclusion
Design	Qualitative studies or mixed‐method studies where the qualitative data could be extracted. Extracted data were related to the experience of involving a carer in therapy.	Studies that only reported on quantitative findings.
Participant	The client was an individual with an intellectual disability aged 18 or over.	Where the client has a diagnosis of autism without the presence of an intellectual disability, or if the client has a diagnosis of dementia or cognitive decline.
Intervention	Psychological therapy was offered to treat a mental health difficulty for someone with an intellectual disability and involved a caregiver. A carer was operationalised as anybody who supported the person with an intellectual disability including family, paid carer, or support staff.	Therapies designed to work with the system or carer only (i.e., carer interventions, family therapy, systematic therapy). Caregiver involvement in the research was purely as an informant rather than being present in therapy. Treatment or intervention was not treating mental health (e.g., physical health intervention or psychosocial intervention).

**FIGURE 1:**
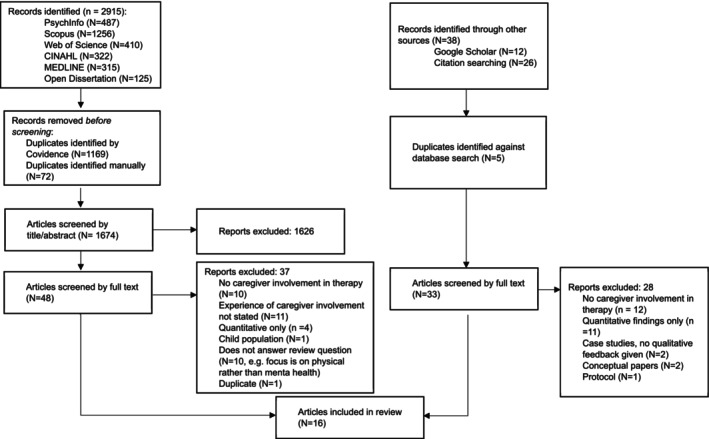
PRISMA flowchart showing the selection of papers.

To ensure no poorly indexed papers were overlooked, a Google Scholar search was conducted on 4 July 2024 using search terms of ‘supporter involvement in psychological therapies intellectual disabilities’ and ‘psychological therapy for individuals with an intellectual disability’. Full text of articles identified as potentially relevant based on title was obtained from the first 10 pages of results. Searching was discontinued at this point as no new relevant papers were being identified (see Figure 1 for PRISMA flowchart).

### 2.3 Quality Appraisal

Critical appraisal is considered an essential component of a systematic review as it can offer the researcher a deeper understanding of the included studies (Cherry et al. 2024). It has previously been used as a way of excluding studies from a review to prevent bias (Atkins et al. 2008). However, within the current review, no study was excluded based on appraisal. It is recognised that appraisal itself can be a subjective process and all studies can provide data which may deepen the interpretation. Additionally, reporting can be constrained by word counts for specific journals. Instead, it is more beneficial to examine papers with a critically reflective lens to extract any potential value (Thorne 2022).

An extended version of the Critical Appraisal Skills Programme qualitative checklist (CASP; Critical Appraisal Skills Programme 2018) was used. This version has been used within previous research using meta‐ethnography exploring the experiences of multiple stakeholders (Poyser and Tickle 2019). Each study was assessed by the first author and quality was rated for each item from zero if not met, one if it was unclear or not fully met and two where definitely met. A random selection of eight studies (50%) was independently assessed by the second author using the same rating scale. Out of 144 ratings, three (2%) were discrepant, by one point each. For these three disagreements, the more critical rating was used for the analysis.

### 2.4 Data Extraction

Key information from each study was extracted by the first author using data extraction templates on Microsoft Word. Information extracted included: study design, type of intervention, type of carer (family or professional), how the carer was involved, whether any adaptations were made to support the client during the interview, key findings, conclusions and limitations.

### 2.5 Synthesis

First and second‐order constructs relevant to answering the review question were entered verbatim into Microsoft Excel with the reviewer's initial interpretations. When reporting findings, most studies that interviewed more than one group separated the findings out into ‘client theme’, ‘carer themes’ or ‘therapist themes’. This was to ensure that no voice was lost when reporting. The current review compared these themes and synthesised together similarities and differences amongst stakeholders and when these may occur to generate an overall understanding of experience.

Common and re‐occurring concepts were identified whilst also considering study characteristics and quality to identify potential differences and similarities. The constructs were compared within and between studies through the processes of reciprocal and refutational translation until the development of third‐order constructs. Finally, the similarities and differences were put together within one interpretation and demonstrated through the line of argument synthesis.

This review outlines the process of developing third‐order constructs through reciprocal and refutational translation before outlining the line of argument. A line of argument is developed through constant comparison of the similarities and differences between third‐order constructs (Sattar et al. 2021). It can bring together different aspects of a topic into a new interpretation (Luong et al. 2023). This makes it helpful in bringing together the perspectives of different groups (Sattar et al. 2021). Therefore, this approach is particularly useful for answering the current review question.

## 3 Results

Sixteen studies were included in this review; see Table 2 for the characteristics of each study and their key findings. At least 243 participants were included amongst the 16 studies (100 people with an intellectual disability, 101 carers, and 42 therapists). This number is not exact as the number of people interviewed was not stated in one study (7). In these cases, it was assumed that all participants also provided qualitative feedback.

**TABLE 2:** Study characteristics.

Study number, author (year), location, source	Aims	Study design	Intervention and caregiver involvement	Sample of participants involved in qualitative part of study	Qualitative data collection method and analysis	Quality appraisal score (max. 36)	Key findings
1. Crossland et al. (2017) United Kingdom (UK) Database	To evaluate a community‐run Dialectic Behaviour Therapy group	MM	18‐week Dialectic Behaviour Therapy (DBT) group Specific involvement is not clear.	Four people with a mild intellectual disability (three female) Age 24–48 White British (3) Other white (1) Three carers (all female) Paid carer (3) Therapist N/I	SSI TA	20	Carers helped with recall. Those without a carer present did not use skills outside the group. Personal benefits to carer. Attending group took carers away from other clients.
2. Douglass et al. (2007) UK Citation searching	To investigate the effectiveness of a cognitive‐behavioural group intervention for anxiety	MM	12‐week Cognitive Behaviour Therapy (CBT) group All clients were required to attend with a carer. Carer involvement was purely supportive within and between sessions.	Six people with an intellectual disability (borderline‐moderate; four females) Aged 22–65 Ethnicity N/S Six carers (sex N/S) Family carer (2) Paid carer (3) Other professional (1) Therapist N/I	SSI for person with intellectual disability Post‐intervention questionnaire for carer Analysis N/S	13	Carers developed a better understanding of anxiety and ways of supporting the person with an intellectual disability. Difficulties when there was a lack of consistency between carers attending sessions.
3. Giannaki and Hewitt (2021) UK Database	To evaluate the effectiveness of a CBT group for adults with learning disabilities who present with anxiety	MM	Seven‐week CBT group for anxiety Carers involved to support generalisation of new skills.	Four people with a mild intellectual disability (3 female) Age N/S White British (4) Four carers (two female) Family carer (3) Paid carer (1) Therapists: N/I	SSI TA	25	One client enjoyed having their carer present. Carers saw their role as helpful and active. Carer involvement helped to generalise learning outside the group.
4. Gillooly et al. (2024) UK Google search	To identify how acceptable the modified BeatIT2 intervention is for adults with severe intellectual disabilities	MM	Behavioural activation for adults with severe and profound intellectual disability and depression (BeatIT2) Carer acted as informant and attended therapy sessions.	People with intellectual disability N/I Five carers (33% of overall sample; four women) Paid carer (2) Family carer (2) Other professional (1) Three therapists	SSI FA	30	Carers and therapists worked in partnership. Carers could provide knowledge about the person with intellectual disabilities and improved their knowledge on mental health. Boosted carers self‐efficacy. Staff inconsistencies and lack of investment from carers was an issue.
5. Griffith et al. (2019) UK Database	To explore how the ‘soles of the feet’ intervention was experienced by people with an intellectual disability, their carer and the therapist	Qual	Six‐session 1:1 mindfulness‐based intervention based on the ‘soles of the feet’ meditation practice Carer actively engaged in the sessions and provided support at home.	Seven people with a mild–moderate intellectual disability (50% of sample; four female) Age 22–51 Ethnicity N/S Six carers were interviewed (75% sample; four female) Family carer (2) Paid carer (3) Other professional (1) Five therapists (50% sample)	SSI TA	28	Carer involvement only mentioned by therapist participants. Carer involvement only beneficial for one therapist. Others said no or negative impact.
6. Haddock and Jones (2006) UK Database	To begin to develop a consensus of ways to engage people with an intellectual disability in CBT	Qual	CBT Type of carer involvement not specifically outlined.	People with an intellectual disability or carers N/I Eight therapists	Questionnaire Qualitative content analysis	20	Carers instrumental in supporting homework and generalise work outside therapy. Success was more likely if carers understood CBT and what therapy involved. Issues around confidentiality and not giving clients a private space to discuss their difficulties openly.
7. Jones and Finch (2020) UK Database	To examine a small group of individuals undertaking an 8‐week group intervention	MM	Eight‐week group intervention incorporating mindfulness and relaxation techniques.	Nine people with a mild intellectual disability. Gender age and ethnicity N/S Two people with an intellectual disability attended with a carer, one of which attended with a different carer each week. Caring role N/S Carer and therapist N/I	Post‐group interviews Analysis N/S	13	Carer involvement allowed information to be generalised.
8. Jones et al. (2021) Canada Database		MM	12‐session DBT group Carers attended the sessions and acted as informants within the research.	15 people with a mild–moderate intellectual disability (75% sample) Of sample involved in intervention (12 female) Ages: 22–52 Ethnicity N/S 38 carers Family carer (2) Paid carer (32) Other professional (4) Therapist N/I	SSI TA	24	Involvement increased carer understanding of the client and taught them new skills. Difficulty passing on information and learning to other workers not involved in group.
9. Knight et al. (2019)^a^ UK Google searching	To explore the experiences of adults with intellectual disabilities randomised into one of two interventions for depression	Qual	Behavioural activation (BeatIT; 15) or Guided Self Help (10) To accompany them to therapy and provide care for a minimum of two‐hours per week.	25 people with a mild–moderate intellectual disability (15.5% sample; 17 female) Ages: 21–66 Ethnicity N/S Therapists and carers were interviewed in other studies (see studies 10 and 11)	SSI FA	29	Carer involvement put the person at ease. Carers supported within and between sessions. Some clients found it unhelpful as they were unable to talk about some things with carer present.
10. Scott et al. (2019)^a^ UK Database	To explore the experiences of carers supporting adults with intellectual disabilities randomised into one of two interventions for depression	Qual	Behavioural activation (BeatIT) or a Guided Self Help To accompany them to therapy and provide care for a minimum of two‐hours per week.	21 carers Family carer (7) Paid carer (14) Therapists and people with an intellectual disability were interviewed in other studies (see studies 9 and 11)	SSI FA	27	Carers built their understanding of the person they supported. Was harder to encourage between session work if carer did not live with client.
11. Smith et al. (2021)^a^ UK Database	To explore the experiences of therapists delivering therapy to adults with an intellectual disability	Qual	Behavioural activation (BeatIT; 15 35%) or a Guided Self Help (11; 32%) To accompany them to therapy and provide care for a minimum of two‐hours per week.	Carers and people with an intellectual disability were interviewed in other studies (see studies 10 and 11) 26 therapists; roles included: Nurse (17) Assistant psychologist (4) IAPT practitioner (2) Occupational therapist (3)	Focus groups and one individual interview using SSI guide FA	26	Therapists found carers useful when they were engaged and if they knew the person well. Experience of involvement was impacted by carer negativity. Reports of carer involvement impacted the therapeutic relationship between therapist and client.
12. Kroese et al. (2014) UK Database	To investigate the views of support staff about the purpose, content, and outcome of CBT	Qual	Nine sessions individual CBT Caregiver involvement was variable between attending all sessions to attending none.	People with intellectual disabilities and therapist N/I Nine carers interviewed (81%) Roles included: Paid carer (4) Other professional (5)	SSI before therapy and after therapy. TA	27	Before CBT, carers saw their role as supportive. They wanted to be more involved but felt confidentiality issues got in the way. After CBT there was little change but more recognition about how they can help increase the person's confidence and be the link with the outside world.
13. Kroese et al. (2016) UK Citation search	To report on an initial attempt to implement a manualised trauma‐focused CBT intervention adapted for people with an intellectual disability	MM	12‐session trauma‐focused CBT group Clients were able to attend with a carer if they wanted.	Five people with an intellectual disability were interviewed (50% group completers; three female) Aged 21–46 Ethnicity N/S Carers and therapists N/I Carer role N/S	SSI IPA	23	Carers facilitated attendance by reducing anxiety. Present of a male carer in group was triggering for some clients. Inconsistency of carer attendance impacted continuity of group. Ethical issues were raised regarding carer attendance.
14. Stimpson et al. (2013) UK Citation search	To explore the expectations and experiences of staff acting as lay therapist for a CBT group.	Qual	CBT group intervention for anger delivered by ‘lay therapists’ Support staff from the day service involved by delivering the intervention.	People with an intellectual disability N/I The carer also acted as therapist in this study. Nine carers (60%; four females) Carer role involved: Paid carer (7) Other professional (2) Time working in ID services: 9 months to 24 years.	SSI IPA	25	Carers were given opportunities to develop knowledge and learn new skills. Carers felt they were closer to the person in the group with greater trust and stronger bonds.
15. Thom et al. (2023) USA Database	To describe a novel virtually delivered group‐based adapted CBT treatment for general anxiety disorder in adults with William's Syndrome.	MM	12‐session virtually delivered CBT group for William's syndrome and anxiety Fourth session from each module was attended by ‘therapy partner’.	Four people with a mild–moderate intellectual disability (two female) Age: 31–33 Ethnicity N/S Information on carer N/S Caring role N/S Therapist N/I	Individual exit‐interview Analysis N/S	15	Taking part equipped carer with new ways to respond to anxiety.
16. Woolfall (2018) UK Database	To explore the experiences of adapted DBT programmes within community intellectual disability services.	Qual	DBT programme some clients attended with a carer.	Six people with a mild intellectual disability (three females) Age: 23–54 Ethnicity N/S Carer and therapist N/I Caring role N/S	SSI IPA	31	Carers helped with skill consolidation. Carer involvement hindered independence.

Abbreviations: IPA = Interpretative Phenomenological Analysis; MM = mixed methods; N/I = not interviewed; N/S = not stated; Qual = qualitative; SSI = semi‐structured interview; TA = thematic analysis.

^a^ These three studies are linked as they emerged from the same randomised controlled trial (Jahoda et al. 2017).

### 3.1 Quality Appraisal

The overall quality ratings for the included studies ranged from 13 to 31 out of a maximum score of 36 (for further information see Data S1). Study quality was also assessed on additional factors relevant to the research question (see Data S1). However, these factors did not contribute to a final score as some items were not applicable within some studies.

Overall quality was varied but most were low. The lowest‐scoring studies (2, 7) were small‐scale, mixed‐method evaluations with a focus on quantitative data, using post‐intervention interviews or questionnaires for qualitative feedback. The highest scoring study (16) was a thesis paper which will have had to adhere to strict reporting requirements and have a larger word count than many journal articles allow.

No study explicitly reported data saturation, likely due to qualitative studies typically being drawn from small samples and data saturation often not being possible. Two studies (9, 10) mentioned selecting participants for diversity of participant characteristics, although this selective sampling method may have contributed to bias.

Only three studies (3, 5, 14) explicitly reflected on the role of the researcher and the potential for bias. Despite this, many of the studies were service evaluations or small‐scale research where the author was also involved in the delivery of the intervention and was therefore likely to be prone to bias.

Ethical standards varied across studies. Most reported participant consent, but higher‐scoring studies (2, 13, 16) detailed confidentiality, withdrawal rights, and ensured participants with intellectual disabilities understood these principles. This is crucial for the population, and the lack of such details in many studies is problematic.

Only three studies (5, 7, 13) explicitly adapted the interview process for people with intellectual disabilities, such as offering prompts (13) or adapting questions (5). However, these adaptations were not always effective; in study 5, fatigue led to withdrawal from the interview in 50% of participants following completion of outcome measures. Furthermore, study 7 made adaptations by encouraging clients to attend post‐group interviews with a carer to assist communication and honesty which may have prevented negative comments on carer involvement. Consequently, even in studies which considered a need for adaptation, findings are still prone to bias.

### 3.2 Synthesis

The meta‐ethnography identified five third‐order constructs. Table 3 outlines these five constructs, including the subthemes of constructs 1, 2, and 4, and indicates which studies contributed to the development of these themes.

**TABLE 3:** Third‐order themes developed from synthesis.

Third‐order theme, subtheme	Study number															
	1	2	3	4	5	6	7	8	9	10	11	12	13	14	15	16
Safety																
Confidentiality						^a^			^a^		^a^	^a^				^a^
Safety in sessions									^a^			^a^	^a^			^a^
Therapeutic process																
Within sessions	^a^			^a^	^a^				^a^		^a^					
Between sessions	^a^	^a^			^a^	^a^	^a^	^a^		^a^	^a^	^a^	^a^	^a^		^a^
Relationships				^a^		^a^		^a^	^a^	^a^	^a^			^a^		
Personal impact on carer																
Increased knowledge and skills	^a^	^a^	^a^	^a^				^a^		^a^				^a^	^a^	
Emotional impact	^a^		^a^	^a^										^a^		
Improving the experience	^a^				^a^	^a^	^a^				^a^		^a^			^a^

^a^ Study was involved in the development of the theme.

### 3.3 Safety

Reciprocal translation and refutational translation both established the theme ‘safety’. This was split into the subthemes of ‘confidentiality’ and ‘safety in sessions’. Six studies (6, 9, 11, 12, 13, 16) contributed to this theme and are relevant to at least one of the subthemes. Within these, therapists, carers and clients discuss ways in which involving carers can make therapy feel safer but also reduce safety.

#### 3.3.1 Confidentiality

Reciprocal translation developed this subtheme from five studies (6, 9, 11, 12, 16). Two studies (6, 11) from carers' perspectives highlight issues when involving carers in therapy, as it can infringe on the person's privacy. In study 11, therapists noted this challenge, with one reporting a client was ‘embarrassed and didn't want to speak in front of his mum’. This raises questions about the carer's role and whether clients' openness varies depending on the caregiver. From the perspectives of people with intellectual disabilities (9, 16), having a carer present limited their ability to speak openly, due to concerns about needing to ‘watch what I was saying’ (9). Thus, involving carers may hinder the person with intellectual disabilities from discussing their experiences honestly. Four studies (6, 9, 11, 12) focused on individual therapy, where confidentiality is more critical. In group therapy, confidentiality issues may be less prominent as the intervention design inherently lacks private space for discussion. Notably, not all individuals with intellectual disabilities in these studies reported negative experiences with carers present; some had positive experiences.

#### 3.3.2 Safety in Sessions

Reciprocal and refutational translation developed this subtheme from four studies (9, 12, 13, 16). People with intellectual disabilities reported that having their carer present made them feel safer, reducing initial anxiety about therapy (9, 13); this was reinforced by one carer (12). For some, involving carers was positive, aiding their engagement in therapy; however, others found it demeaning, as it reduced their independence (9, 16). In study 13, some participants considered carer involvement unsafe, especially with male carers, which was triggering. This study, focusing on PTSD, suggests that the acceptability of involving carers depends on the individual's trauma experiences.

### 3.4 Therapeutic Process

Thirteen studies (1, 4, 5, 6, 7, 8, 9, 10, 11, 12, 13, 14, 16) were compared through the process of reciprocal and refutational translation in the development of this theme. It focuses on the experience of carer involvement and how it may influence the therapeutic process.

#### 3.4.1 Within Sessions

Five studies showed that carer involvement in therapy can assist the therapeutic process (1, 4, 5, 9, 11). Clients' experience was improved when carers helped them understand therapeutic concepts (1, 9); however, this was only the case when carers understood the content themselves (5). Additionally, therapists (4, 11) spoke about carers facilitating communication. Negative caregiver attitudes in sessions were found to be problematic to the therapeutic process and therefore it was suggested that the relationship of the carer and the client was particularly important as the carer also needed to be motivated and willing to do the work (4, 5, 11). Inconsistent carer attendance was also a challenge (4, 5, 11) and negatively impacted the experience. Therapist interviews highlighted these issues, likely because carers who were interviewed were likely to be more motivated.

#### 3.4.2 Between Sessions

Many studies highlighted the benefits of carer involvement in helping people with intellectual disabilities generalise therapy knowledge between sessions (1, 2, 6, 7, 10, 12, 13, 14, 16). Studies included within this review described interventions based on skill development, so clients used carers to support homework tasks and apply learning outside therapy, which was endorsed by clients, carers and therapists alike (1, 6, 7, 10, 12, 13, 14, 16). Clients without carers found it harder to use skills outside therapy (1, 7), suggesting carer involvement would have been beneficial and improved the experience. Two studies (1, 2) indicated clients without consistent carers struggled more with retaining and generalising information. Furthermore, there appeared to be systemic barriers, which made generalisation between sessions difficult, particularly when carers changed frequently (2, 5, 7) or many carers were involved in the clients' care (4, 8, 11). Carer negativity and motivation also made it less likely for people with intellectual disability to continue the work outside therapy (4, 11).

### 3.5 Relationships

Reciprocal and refutational translation led to the development of the theme ‘relationships’, which was represented in seven studies (4, 6, 8, 9, 10, 11, 14). Three studies commented on the working relationship between therapist and carer (4, 10, 11). Carers, initially knowing the clients better, shared valuable insights. This was particularly pertinent in study 4, involving individuals with severe intellectual disabilities, where carer insights were crucial. However, therapists reported weaker relationships with less motivated carers, who questioned their involvement and sometimes hindered the therapeutic process (11). In this study (11), therapists sometimes felt that the presence of carers restricted the therapeutic relationship.

Improved relationships between client and carer were also noted (6, 9, 10, 14), often stemming from carers' better understanding of the clients through therapy sessions. Such understanding enabled carers to support clients more effectively. Yet, these observations might be biased, as more engaged carers were likely interviewed in studies 10 and 14. Additionally, in study 14, some carers felt frustrated when clients couldn't apply group session learnings.

Only one study (9), interviewing people with intellectual disabilities, commented on the carer–client relationship. Clients saw therapy as a ‘joint venture’, appreciating the mutual benefits giving a sense of them being on an equal footing. Whilst positive for clients, this level of carer involvement might detract from the focus on the person with an intellectual disability, as noted in study 6. Additionally, one study (8) investigating a group intervention, commented on the development of relationships between other caregivers.

### 3.6 Personal Benefit to the Carer

Reciprocal translation led to the development of this theme. Most studies (1, 2, 3, 4, 8, 10, 14, 15) that interviewed carers commented on some of the personal impacts that being present in sessions had on them. The theme split into subthemes, ‘increased knowledge and skills’, and ‘emotional impact’.

#### 3.6.1 Increased Knowledge and Skills

Many carers commented on how being involved in therapy helped increase their knowledge about mental health (2, 4, 10, 14) as well as helping them learn new skills for coping (1, 3, 8, 10, 15). This was a personal benefit for themselves and for their role as a carer. Many carers who were in a paid supporting role also hoped to use their increased knowledge for future clients (1, 2, 10, 14), suggesting that there was also an occupational benefit of their involvement and benefits for future clients.

#### 3.6.2 Emotional Impact

Carers also commented on the emotional benefits of being involved in therapy (1, 3, 4, 14). For some, they were able to relate to the content of therapy themselves meaning they were able to apply the skills from therapy to help them deal with problems in their own life (1, 3) Additionally, there were reports of feeling more confident within their role as a carer (4, 14).

### 3.7 Improving the Experience

Seven studies (1, 5, 6, 7, 11, 13, 16) commented on ways to improve carer involvement in therapeutic interventions. Some suggested offering additional ‘top‐up’ sessions for client and carers to help them maintain their skills beyond therapy. Others (5, 7, 11, 13) suggested specific training sessions for staff prior to the intervention, to provide them with information about therapy and their role in it, hoping to increase motivation and engagement from the carer and to support their ability to help individuals with an intellectual disability understand therapy. Other studies (6, 16) highlighted the importance of client choice in deciding whether to involve their carer or offering the client time during sessions on their own without the carer (11) to enable them to talk more openly. To overcome more systemic issues regarding carer consistency, it was suggested that a conversation with service managers about the importance of allocating the same caregiver may be required (13).

### 3.8 Line of Argument Synthesis

Third‐order constructs show the varied experiences regarding carer involvement in therapy. These were due to differences in the level of engagement and motivation from the carer and systemic challenges such as inconsistency in carer involvement. Individual differences were also a factor with some people with an intellectual disability enjoying their carer being present as it helped with their anxiety, whilst others would have preferred the independence to attend alone. The synthesis did however demonstrate that there were many positives to involving caregivers in helping with skills consolidation and generalisation outside therapy and improved relationships through increased carer knowledge and understanding and suggested that carers who have a closer personal relationship with the person may be more motivated to spend time with them and thus more suitable. It has also highlighted the importance of offering the person with intellectual disabilities a choice of whether to involve their carer or not.

## 4 Discussion

This review synthesised qualitative literature on the experiences of involving carers in therapy from the perspectives of therapists, carers and individuals with intellectual disabilities. Whilst guidance recommends carer involvement (Beail 2017; NICE 2016), and previous reviews note it as common (Surley and Dagnan 2019; Tapp et al. 2023), this review uniquely examined qualitative accounts of the experience. Five third‐order constructs were developed, revealing mixed experiences within and between studies.

Caregivers in the current review primarily had a supportive role, assisting with homework and facilitating communication. Previous research reports that this is an advantage of carer involvement (Dagnan et al. 2023). Perhaps for these reasons, carer involvement has been suggested to lead to greater therapy success (Rose et al. 2005). However, the current review found that carer motivation and engagement varied, sometimes negatively impacting client understanding. Frequent carer changes and poor communication amongst staff also made carer involvement more inconvenient than helpful. Thus, whilst previous research highlights benefits, the practical experience is often different, aligning with concerns reported by Dagnan et al. (2023). It may be possible that this occurs less frequently when it is an informal carer, or family member, rather than a paid carer. The studies included within the current review often reported what type of carer was involved; however it was not possible to distinguish whether results differed between formal and informal caregivers. It is likely that family members may show greater motivation and engagement and are less likely to change each week. Future research may explore differences in outcomes between paid and family carers.

Only one study within the current review explored an intervention that was carer‐led (Stimpson et al. 2013). This was found to be a positive experience in contrast to previous literature which recruited parents as co‐therapists reporting difficulties (Lundkvist‐Houndoumadi et al. 2015). These findings add to previous reviews (Hithersay et al. 2014) on carer‐led interventions, but add insights from individuals with intellectual disabilities. Wherever carers have been interviewed, they mostly reported positive experiences which led to the development of the construct focusing on the positive impact on carers. However, this should be treated with caution as it is likely that, within these studies, only the carers who were the most engaged and motivated consented to participate in follow‐up interviews, which may well have led to a response bias.

Most studies in this review focused on anxiety, depression or emotion regulation difficulties. Only one study targeted post‐traumatic stress disorder (Kroese et al. 2016), but it emphasised skill development over trauma processing. Carer involvement has been considered feasible in previous trauma processing therapies, specifically EMDR (Schipper‐Eindhoven et al. 2024). However, the current review did not find comments on carers' feelings about hearing the person they care for discuss their difficulties, likely due to the skills‐based nature of the included interventions. It was noted that clients sometimes struggled to speak openly in front of carers, indicating potential issues with carer involvement that warrant further research, particularly for sensitive topics. The clinical implication is to remember whom the therapy should benefit, and therefore considering the importance of the client feeling able to talk openly.

Apart from one study (Gillooly et al. 2024), where stated, all people with an intellectual disability were in the mild–moderate range. This may be related to difficulties in gaining informed consent for this population. Despite this, carers and therapists reported similar experiences across studies, regardless of disability severity. However, this specific study did not contribute to the third‐order construct relating to confidentiality or safe spaces for clients to talk, possibly because the person with an intellectual disability was not interviewed, meaning the perspectives of individuals with a severe intellectual disability are unrepresented. However, having a severe intellectual disability can limit the ability to comment due to limited language skills, and the DSM‐5 notes the requirement of ongoing support (APA 2013). Consequently, carer involvement may be necessary.

The quality of the included studies was generally low, consistent with previous reviews on therapy for individuals with intellectual disabilities (Tapp et al. 2023). Future research could be improved by specifying potential biases within the study to help readers assess the validity of papers. Furthermore, due to the fact that people with an intellectual disability are a vulnerable population, it is important that researchers explicitly state ethical practices, and any adaptations that they made to ensure the person understood what the study and their role involved. These adaptations may include those suggested in previous research (Drozd et al. 2021). Only a few studies explicitly stated that adaptations were made for interviews. However, this may be because of the word limits when publishing journals, or because researchers may not feel adaptations are required within typical interviewing procedures. Regardless, without adaptations, it may be that some people may not have understood the questions asked, leading to unreliable findings. Furthermore, including a carer in the interview process would also be counterintuitive if wanting to understand whether this is a feasible or acceptable adaptation of therapy.

### 4.1 Strengths and Limitations

The search strategy used aimed to capture and identify a wide range of studies. Using citation searching allowed an additional five studies to be identified. This was particularly beneficial for this review and ensured additional data relating to experiences was reported on.

Additionally, it was decided not to exclude studies based on language or date to avoid missing potentially important data. However, it is notable that the oldest study included was from 2006. Clinical guidance and policies have changed considerably since then with NICE guidance being published in 2016. The importance of involving people with an intellectual disability in research has been highlighted (Beighton et al. 2019) and it is noteworthy that the three oldest studies in the review did not interview the person with the intellectual disability. It is likely that more recent studies are more representative of current practice.

Despite not excluding studies based on date, it has been noted by the authors that ‘mental retardation’ was not used within the search terms despite this being a term frequently used in many old studies. With this in mind, it is possible that some older studies may have been inadvertently missed.

Only three studies reported ethnicity demographics for the person with an intellectual disability; where this was stated, the majority were White British. Future research should improve on the reporting of key participant demographics. Furthermore, all but two studies (Jones et al. 2021; Thom et al. 2023) were conducted in the United Kingdom, with the remainder in other Western countries. Other cultures may hold different experiences of caregiver involvement, especially those with greater emphasis on caring within a community. Thus, the findings from this review are not generalisable to other ethnicities or cultures and future research is needed to explore differences.

A limitation of the current review is that screening, extraction and synthesis were conducted solely by the first author. Therefore, findings are subjective and driven by the author's interpretation. However, interpretations from synthesis were discussed with the second author to ensure face validity.

### 4.2 Research and Clinical Implications

This review highlights that, although many studies on mental health interventions for people with intellectual disabilities involve a carer, few inquire about the experience of having the carer present. Most studies focus solely on the effectiveness of interventions and do not address the participant experience, or they treat it as a secondary aspect rather than the primary focus. Future research should more closely examine the acceptability of carer involvement in therapy for individuals with intellectual disabilities. It may be interesting to explore whether experiences differ depending on whether it is a family, or paid carer involved.

This review identified that most interventions used in the studies that commented on carer involvement, were skills‐based, rather than exploratory, therapies. Therefore, future research should investigate the experience of involving carers in therapy where the individual may be required to discuss the impact of past events on current behaviour.

NICE guidance (2016) recommends collaborating with individuals and their families or caregivers when delivering psychological interventions for people with intellectual disabilities. However, this review indicates that carer involvement can sometimes impede the therapeutic process and interfere with the individual's privacy, despite its potential benefits. Therefore, further specific guidance is needed to optimise carer involvement and enhance the experience for all parties. The following guidance has been suggested based on the construct ‘Improving the experience’ and the line of argument synthesis:

- Where the person with an intellectual disability has been assessed to have the cognitive and language ability to make and communicate an informed decision, they should be offered the choice as to whether they would like their caregiver to be present in therapy.
- If carers are not present, they should be offered resources and training about therapy and ways they may be able to support the person between sessions.
- Where caregivers are present, the person with an intellectual disability should be offered time alone with the therapist to discuss anything they do not want to share in front of their carer.
- When involving carers, their role and relationship with the person with an intellectual disability should be considered. Carers should have a good level of engagement, motivation and investment within therapy and be in a role where they are able to support the person between sessions.
- Where possible, the same carer should attend each session to ensure consistency. If the person lives in supported accommodation, further resources on therapy and how to support the person could be offered to other staff involved in the person's care.

## 5 Conclusions

This review has highlighted the varied experiences reported by clinicians, carers and people with intellectual disability regarding carer involvement in therapy. There are several reasons that may influence the experience of carer involvement. Clinical implications suggest the importance of patient choice about whether and how to involve the carer, and where carers are involved, they should be well informed about the intervention and what their involvement entails. Future research is needed to determine how carer involvement is experienced in therapies that are more explorative regarding sensitive topics, such as traumatic experiences.

## Ethics Statement

The authors have nothing to report.

## Conflicts of Interest

The authors declare no conflicts of interest.

## Supporting information


**Data S1:** Supporting information.
