# Supplementary material for: Involving Carers in Therapy for Adults With Intellectual Disabilities: A Systematic Review of Client, Carer and Therapist Perspectives
Source: J Appl Res Intellect Disabil. 2025 Nov 19;38(6):e70152. doi: 10.1111/jar.70152 (PMC12628289; doi:10.1111/jar.70152)
Supplement: Supplementary file 1 — Data S1: Supporting information. [file JAR-38-e70152-s001.docx]

Quality Appraisal Table

|  | Study Number | | | | | | | | | | | | | | | |
| --- | --- | --- | --- | --- | --- | --- | --- | --- | --- | --- | --- | --- | --- | --- | --- | --- |
|  | 1 | 2 | 3 | 4 | 5 | 6 | 7 | 8 | 9 | 10 | 11 | 12 | 13 | 14 | 15 | 16 |
| Quality Criteria |  |  |  |  |  |  |  |  |  |  |  |  |  |  |  |  |
| 1. A clear statement of aims | 2 | 2 | 2 | 2 | 2 | 2 | 1 | 2 | 2 | 2 | 2 | 2 | 0 | 2 | 2 | 2 |
| 1. Qualitative methodology is appropriate to capture subjective experience | 2 | 1 | 1 | 2 | 2 | 2 | 1 | 2 | 2 | 2 | 2 | 2 | 2 | 2 | 1 | 2 |
| 1. Was the research design appropriate to address the aim? | 1 | 0 | 0 | 2 | 1 | 1 | 0 | 1 | 2 | 2 | 2 | 1 | 1 | 2 | 0 | 2 |
| 1. Was the recruitment strategy fully explained | 1 | 2 | 2 | 2 | 2 | 2 | 2 | 2 | 2 | 2 | 1 | 2 | 1 | 2 | 2 | 2 |
| 1. Was the recruitment strategy appropriate to meet the aims? | 1 | 1 | 1 | 2 | 1 | 1 | 1 | 2 | 1 | 1 | 1 | 1 | 1 | 1 | 2 | 2 |
| 1. Was it clear how the data was collected (e.g. interviews)? | 2 | 2 | 2 | 2 | 1 | 2 | 1 | 2 | 2 | 2 | 1 | 2 | 2 | 2 | 1 | 2 |
| 1. Form of data clear (e.g. tape recordings or video)? | 0 | 0 | 2 | 2 | 1 | 2 | 0 | 0 | 2 | 2 | 2 | 2 | 0 | 2 | 0 | 2 |
| 1. Discussion of data saturation | 0 | 0 | 0 | 0 | 0 | 0 | 0 | 0 | 1 | 1 | 0 | 0 | 0 | 1 | 0 | 0 |
| 1. Critical examination of researcher role and potential for bias | 0 | 0 | 2 | 0 | 2 | 0 | 1 | 0 | 0 | 0 | 0 | 0 | 0 | 2 | 0 | 0 |
| 1. Sufficient details to assess ethical standards | 0 | 2 | 1 | 1 | 1 | 0 | 1 | 1 | 1 | 1 | 1 | 1 | 2 | 0 | 1 | 2 |
| 1. Ethics committee approval mentioned | 2 | 0 | 2 | 2 | 2 | 0 | 0 | 2 | 2 | 2 | 0 | 2 | 2 | 0 | 2 | 2 |
| 1. In depth description of analysis | 0 | 0 | 2 | 1 | 2 | 0 | 0 | 1 | 1 | 1 | 2 | 1 | 1 | 1 | 0 | 2 |
| 1. Sufficient evidence presented to support themes | 1 | 0 | 1 | 2 | 2 | 1 | 1 | 1 | 2 | 2 | 2 | 1 | 2 | 2 | 1 | 2 |
| 1. Contradictory data taken into account | 2 | 0 | 1 | 2 | 2 | 2 | 1 | 1 | 2 | 2 | 2 | 2 | 2 | 1 | 0 | 2 |
| 1. Clear statement of findings | 2 | 1 | 2 | 2 | 2 | 2 | 1 | 2 | 2 | 2 | 2 | 2 | 2 | 2 | 0 | 2 |
| 1. Discussion of credibility of findings (e.g. triangulation, more than one analyst) | 1 | 1 | 1 | 2 | 1 | 0 | 1 | 2 | 2 | 2 | 2 | 2 | 2 | 2 | 0 | 1 |
| 1. Clear contribution to existing knowledge or understanding | 2 | 0 | 2 | 2 | 2 | 1 | 1 | 2 | 2 | 2 | 2 | 2 | 2 | 1 | 2 | 2 |
| 1. New areas of research identified | 1 | 1 | 1 | 2 | 2 | 2 | 0 | 1 | 2 | 0 | 2 | 2 | 1 | 0 | 2 | 2 |
| Total | 20 | 13 | 25 | 30 | 28 | 20 | 13 | 24 | 29 | 27 | 26 | 27 | 23 | 25 | 15 | 31 |

0 = Not met
1 = Unclear or not fully met
2 = Clearly met

Extended version of the Critical Appraisal Skills Programme qualitative checklist (Poyser & Tickle, 2019).

Review specific quality appraisal

|  | Study Number | | | | | | | | | | | | | | | |
| --- | --- | --- | --- | --- | --- | --- | --- | --- | --- | --- | --- | --- | --- | --- | --- | --- |
|  | 1 | 2 | 3 | 4 | 5 | 6 | 7 | 8 | 9 | 10 | 11 | 12 | 13 | 14 | 15 | 16 |
| Were adaptations made for interviewing the client? | 0 | 0 | 1 | NA | 2 | NA | 2 | 0 | 0 | NA | NA | NA | 2 | NA | 0 | 1 |
| Were carers present every session? | 1 | 2 | 2 | 2 | 2 | NA | 0 | 2 | 2 | 2 | 2 | 0 | 1 | 2 | 0 | 1 |
| Was it the same carer each session? | 2 | 1 | 2 | 2 | 0 | NA | 0 | 0 | 0 | 0 | 0 | 0 | 1 | 1 | 1 | 1 |
| Total | 3 | 3 | 5 | 4 | 4 | NA | 2 | 2 | 2 | 2 | 2 | 0 | 4 | 3 | 1 | 3 |

NA = Not applicable
0 = Not met
1 = Unclear or not fully met
2 = Clearly met
